# Incidence of Extended Spectrum β-Lactamase Genes (ESBLs) among community and health care infection in Mansoura University Hospital, Egypt

**DOI:** 10.1186/s12866-025-04030-3

**Published:** 2025-05-22

**Authors:** Alaa Aboelnour Badran, Fatma A. Elgayar, Mona K. Gouda, Nancy M. El Halfawy

**Affiliations:** 1 Department of Clinical Pathology, Faculty of Medicine, Mansoura University, Mansoura, Egypt; 2 Department of Botany and Microbiology, Faculty of Science, Alexandria University, Alexandria, Egypt

**Keywords:** Antimicrobial resistance (AMR), Multidrug-resistant (MDR), Extended-spectrum β-lactamases (ESBLs)

## Abstract

**Background:**

Multidrug-resistant (MDR) Gram-negative bacteria pose a significant challenge due to their limited treatment options. The production of extended-spectrum β-lactamases (ESBLs) is an important mechanism of resistance. This study aimed to identify the incidence and characteristics of ESBL-encoding genes (*bla*_CTX-M_, *bla*_TEM_, *bla*_SHV_, and *bla*_OXA_) in MDR isolates.

**Materials and methods:**

A cross-sectional study was conducted from September 2022 to May 2023. ESBL-producing isolates (*n* = 105) out of 412 were recovered from hospitalized and outpatient settings and analyzed. Standard microbiological methods were used for isolates identification, susceptibility testing, and phenotypic ESBL detection. Additionally, *bla*_CTX-M_, *bla*_TEM_, *bla*_SHV_, and *bla*_OXA_ genes were identified using conventional PCR.

**Results:**

Molecular profiling of β-lactamase determinants was conducted via PCR targeting *bla*_CTX-M_, *bla*_TEM_, *bla*_SHV_, and *bla*_OXA_ genes. Among phenotypically confirmed (100%) ESBL producers, 98% harbored one or more target genes, with *bla*_CTX-M_ predominant (81%), followed by *bla*_SHV_ (70.4%), *bla*_TEM_ (62%), and *bla*_OXA_ (30.4%). Carbapenem resistance was higher in ESBL-producing strains compared to non-ESBL strains. Extensively drug-resistant (XDR) isolates were the most common across hospital departments and outpatients.

**Discussion:**

This study highlights the significant prevalence of ESBL genes and multidrug resistance among Gram-negative bacteria. The dominance of *bla*_CTX-M_ and the existence of multiple resistance genes raise concerns about limited treatment options. The findings emphasize the need for stricter antibiotic stewardship and infection control measures to curb the spread of MDR pathogens.

**Conclusion:**

This study provides valuable insights into the alarming incidence of ESBL genes and MDR in Mansoura, Egypt. Continuous surveillance and implementation of effective control strategies are crucial to combat this growing public health threat.

**Supplementary Information:**

The online version contains supplementary material available at 10.1186/s12866-025-04030-3.

## Introduction

The global health crisis of antimicrobial resistance (AMR) has reached alarming proportions, with Egypt experiencing a particularly significant burden. AMR has negative impacts as it is associated with high rates of mortality. In 2019, the country reported a staggering 16,100 deaths directly attributed to AMR, highlighting the urgent need for effective interventions. Extended-spectrum β-lactamases (ESBLs), a class of enzymes that confer resistance to β-lactam antibiotics, have played a pivotal role in this crisis [1, 2].

ESBLs have been rapidly diversifying, with over 300 distinct types identified. Among the most prevalent ESBL genes are CTX-M, SHV, TEM, VEB, GES, PER, TLA, and OXA variants, which exhibit enhanced catalytic activity against ceftazidime, cefotaxime, and ceftriaxone. These enzymes are commonly found in Enterobacteriaceae, particularly *Escherichia coli* and *Klebsiella pneumoniae*, *Acinetobacter*, and *Pseudomonas* species [3, 4].

The dissemination of AMR in Egypt is driven by several factors, including the overuse of antibiotics in human and veterinary medicine, environmental contamination, and inadequate healthcare infrastructure. In addition, poor sanitation and ignorant clinical practice aid in the spread of multidrug-resistant (MDR) microbial strains. These factors contribute to the global spread of resistant bacteria through trade, travel, and migration [5, 6].

A concerning trend is the increasing incidence of ESBL-producing bacteria in community settings. This suggests that antibiotic resistance is no longer confined to healthcare facilities and poses a greater threat to public health. Studies have shown a significant rise in the incidence of ESBL-producing *E. coli* in community settings, from 2.6% in 2001–2005 to 26.4% in 2016–2020. This increase is attributed to human-to-human transmission, food contamination, and global travel [4, 7].

To address this pressing issue, it is crucial to understand the incidence and types of ESBL genes circulating in Egypt. This knowledge will inform targeted interventions to mitigate the impact of AMR and protect public health. Therefore, a comprehensive strategy must be developed to address the challenge of AMR [8, 9].

This study's objective was to determine the prevalence of β-lactamases encoded by the *bla*_CTX-M_, *bla*_OXA_, *bla*_SHV_, and *bla*_TEM_ genes among clinical isolates of Enterobacteriaceae and *Acinetobacter baumannii* obtained from healthcare-associated and community-acquired specimens in Mansoura, Egypt.

## Materials and methods

### Study design

This cross-sectional observational study aimed to investigate the prevalence of β-lactamase genes (*bla*_CTX-M_, *bla*_OXA_, *bla*_SHV_, and *bla*_TEM_) in Enterobacteriaceae and *Acinetobacter baumannii* isolates obtained from clinical specimens at Mansoura University Hospital, Egypt. Between September 2022 and May 2023, 412 non-duplicate Gram-negative clinical isolates were collected from diverse clinical sources within the hospital.

### Study setting

The study was conducted at Mansoura University Hospital, a tertiary care facility in the Dakahlia Governorate, Egypt. Serving a large and diverse patient population, including both inpatients and outpatients, this hospital provided an optimal environment for characterizing the epidemiology of extended-spectrum β-lactamase (ESBL)-producing isolates in clinical settings.

### Clinical isolate collection and processing

Clinical specimens (blood, urine, pus, wound swabs, cerebrospinal fluid [CSF], and respiratory samples) were collected via convenience sampling from inpatient departments and outpatient clinics of Mansoura University Hospital using a standardized protocol. Ethics considerations, including personal data privacy, were considered during all study steps. Blood specimens were inoculated into a BACTEC blood culture bottle at a 1:10 ratio (2.5 mL blood to 25 mL Brain Heart Infusion broth) and loaded into the BACTEC system for incubation at 35–37 °C under aerobic conditions (BD BACTEC™ Automated Blood Culture Systems). Subculturing onto blood agar, chocolate agar, and MacConkey agar (Oxoid, UK) was performed upon visible microbial growth. Moreover, urine specimens were cultured on cysteine lactose electrolyte deficient (CLED) (Oxoid, UK) agar using a calibrated 5 μL disposable loop. Specimens with significant bacteriuria (≥ 10^5^ CFU/mL) were retained for analysis. Other specimens (pus, wound, CSF, respiratory) were processed on blood agar, chocolate agar, and MacConkey agar under aerobic incubation at 35–37 °C for 24–48 h. Blood cultures were extended to 5 days to detect slow-growing pathogens.

### Bacterial identification

Isolates were identified using colony morphology, Gram-staining technique, and biochemical assays, including catalase, oxidase, coagulase, and IMViC (indole, methyl red, Voges-Proskauer, citrate) tests. All were standardized protocols per the Manual of Clinical Microbiology (13 th edition) [10, 11]. Mixed cultures or polymicrobial isolates were excluded. Single colonies were purified by streaking on MacConkey agar plates. Pure isolates were stored at −20 °C in tryptic soy broth (TSB; Oxoid, UK) containing 50% (v/v) glycerol for downstream analyses. Identified isolates underwent antimicrobial susceptibility testing (AST) and genomic DNA extraction for molecular characterization.

### Antibiotic susceptibility testing (AST)

The antibiotic susceptibility testing was conducted using the automated Microbial AST System DL-96 (Zhuhai DL Biotech, China). The following antibiotics were tested: Amoxicillin–Clavulanic acid (20/10 µg), Cefotaxime (30 µg), Ceftriaxone (30 µg), Ceftazidime (30 µg), Gentamicin (10 µg), Amikacin (30 µg), Ciprofloxacin (5 µg), Imipenem (10 µg), Amoxicillin (30 µg), Cephalexin (30 µg), Cefuroxime (30 µg), Co-Trimoxazole (25 µg), Meropenem (10 µg) and Doxycycline (30 µg).

### Phenotypic Identification of ESBLs

Third-generation cephalosporin-resistant isolates were subjected to the double disc synergism test (DDST) and combination disc test (CDST) to confirm ESBL production on Mueller–Hinton agar (Oxoid, UK). The quality control of the tests was performed by the standard strains of *E. coli* (ATCC 25922) and *K. pneumoniae* (ATCC 700603) as negative and positive controls, respectively. Keyhole effect for the DDST and ≥ 5 mm zone enhancement of ceftazidime and clavulanate in comparison to ceftazidime alone for the CDT were considered positive tests for ESBL production [11, 12].

### Extraction of genomic DNA

Genomic DNA was extracted from 1.5 mL of overnight bacterial cultures grown in TSB using the HiPurA Genomic DNA Purification Kit (HiMedia, India), following the manufacturer’s instructions. The concentration and purity of the extracted DNA were assessed via a NanoDrop ND-2000 spectrophotometer (Thermo Fisher Scientific, USA) and 1% (w/v) agarose gel electrophoresis. Following DNA quantification, all DNA samples were stored at −20 °C for further investigation.

### Genotypic detection of ESBL genes

Purified genomic DNA samples were used as a template for PCR amplification of the *bla*_SHV_, *bla*_TEM_, *bla*_CTX-M_, and *bla*_OXA_ genes [8, 13]. Table 1 provides the specific oligonucleotide primers and PCR amplification conditions used in this study. The primers were synthesized by Bio Basic Inc. (Canada) and resuspended in nuclease-free water to a final concentration of 10 pmol/µL. PCR amplification was performed using 2X EasyTaq PCR SuperMix (Transgenbiotech) according to the manufacturer's instructions. Each 25 μL PCR reaction mixture contained 12.5 µL of master mix,1 µL of 1 mM of each primer, 3 µL of target DNA, and 7.5 µL nuclease-free water. PCR reactions were performed in a TECHNE TC-3000 thermal cycler. PCR amplicons were resolved by electrophoresis on a 1% (w/v) agarose gel prepared in 1X Tris–Borate-EDTA (TBE) buffer, and the size of the target fragment was determined. The gel was stained with a 5% (w/v) ethidium bromide solution and visualized using a UV transilluminator.

**Table 1:** Primers and conditions used for the amplification of ESBL genes

Gene	Sequence 5’−3’ F/R	Size (bp)	Annealing Temp (°C)
***bla***_**OXA**_	**bla-OXA F:** AGCAGCGCCAGTGCATCA	708	60
	**bla-OXA R:** ATTCGACCCCAAGTTTCC		
***bla***_**TEM**_	**bla-TEM F:** CCGCTCATGAGACAATAACC	931	58
	**bla-TEM R:** GGTCTGACAGTTACCAATGC		
***bla***_**CTX-M**_	**bla-CTX-M F:** TCTTCCAGAATAAGGAATCCC	909	58
	**bla-CTX-M R:** CCGTTTCCGCTATTACAAAC		
***bla***_**SHV**_	**bla-SHV F:** TGGTTATGCGTTATATTCGCC	868	60
	**bla-SHV R:** GGTTAGCGTTGCCAGTGCT		

### MDR, XDR, and PDR classification

Bacterial isolates were categorized as multidrug-resistant (MDR), extensively drug-resistant (XDR), or pan-drug-resistant (PDR) based on their susceptibility profiles to a broad range of antimicrobial agents, including carbapenems, cephalosporins, aminoglycosides, fluoroquinolones, and sulfonamides.

### Data completeness and accuracy

To ensure data completeness and accuracy, strict adherence to Standard Operating Procedures (SOPs) for microbiological procedures was maintained throughout the study. Clinical specimens were handled with meticulous care from collection to processing. Media, reagents, and antimicrobial discs were checked for expiration dates before use, and the integrity of the culture media was verified for sterility and physical defects.

### Quality Control Measures

This included testing new batches of media and reagents against control strains. For ESBL confirmation, *K. pneumoniae* (ATCC 700603) and *E. coli* (ATCC 25922) were used. By implementing these rigorous quality control measures, we ensured the reliability and validity of the study findings. The incidence of ESBL genes was determined, and the distribution of different ESBL types was analyzed. Additionally, the resistance patterns of the isolates to various antibiotics were evaluated.

## Results

### Study Population and clinical isolates

A total of 412 non-duplicate Gram-negative bacterial isolates were collected from hospitalized patients at Mansoura University Hospital between September 2022 and May 2023. Of these, 105 (25.5%) were identified as extended-spectrum β-lactamase (ESBL) producers by phenotypic methods.

The most common ESBL-producing bacterial species were *Escherichia coli* (39%, *n* = 41) and *Klebsiella pneumoniae* (24%, *n* = 25), followed by *Enterobacter* spp. (13%, *n *= 14), *Acinetobacter* spp. (12%,* n* = 13), *Proteus* spp. (10%, *n* = 10), and *Stenotrophomonas maltophilia* (2%, *n* = 2). The majority of isolates were recovered from respiratory samples (34.6%, *n* = 36), followed by pus (including aspirate) (26.2%, n = 27), urine (23.4%, *n* = 25), blood (11%, *n* = 12), and cerebrospinal fluid (CSF) (4.8%, *n *= 5).

### Demographic and clinical characteristics of patients with ESBL-positive isolates

The study included 105 patients with ESBL-positive isolates. The majority of patients were male (62.9%), with an average age of 52.3 years (Standard deviation: ± 15.4 years). As shown in Table 2, the most common underlying medical conditions were diabetes mellitus (28.6%), chronic obstructive pulmonary disease (COPD) (22.9%), and hypertension (18.1%).

**Table 2:** Demographic Characteristics of Patients with ESBL-Producing

Characteristic	Value
**Gender, n (%)**	
Male	66 (62.9)
Female	39 (37.1)
**Age (years)**	52.3 ± 15.4^a^
**Underlying comorbidities, n (%)**	
Diabetes mellitus	30 (28.6)
Chronic obstructive pulmonary disease (COPD)	24 (22.9)
Hypertension	19 (18.1)
Other conditions	32 (30.5)

^a^Data presented as mean ± standard deviation

Table 3 details the distribution of ESBL-producing isolates across hospital settings and specimen types, with frequencies stratified by intensive care units (general/pediatric) and outpatient departments. Urine was the most predominant specimen source, particularly among outpatients (44.4%), whereas bloodstream was the most frequently collected in the pediatric ICU (58.8%). Pus samples were predominantly from outpatients (32%), while CFS samples were more common in pediatric ICU patients (14.9%) compared to the other groups. Notably, the proportion of ESBL-positive isolates was highest in general ICU patients (40.3%), followed by pediatric ICU (28.1%) and outpatient (12.4%), with a significant overall difference (*p* < 0.001).

**Table 3:** Distribution of isolates across hospital settings and sample types

Sample Type	ICU General *n (%)*	ICU Pediatric *n (%)*	Outpatient *n (%)*	Total *n (%)*	*p*-value
Pus	15 (11.6)	10 (8.8)	54 (32.0)	79 (19.2)	< 0.001
Blood	38 (29.5)	67 (58.8)	0 (0.0)	105 (25.5)	< 0.001
CSF	10 (7.8)	17 (14.9)	0 (0.0)	27 (6.6)	< 0.001
Respiratory	16 (12.4)	11 (9.6)	40 (23.7)	67 (16.3)	< 0.001
Urine	50 (38.8)	9 (7.9)	75 (44.4)	134 (32.5)	< 0.001
Total Samples	129 (100)	114 (100)	169 (100)	412 (100)	–
ESBL-Positive	52 (40.3)	32 (28.1)	21 (12.4)	105 (25.5)	< 0.001

*ICU* Intensive Care Unit, *ESBL* Extended-spectrum β-lactamase, *CSF* Cerebrospinal fluid

### Genetic detection of ESBL genes

The most prevalent ESBL genes were *bla*_CTX-M_, detected in 81% of isolates (*n* = 85), followed by *bla*_TEM_ (62%, *n *= 74), *bla*_SHV_ (70.4%, *n* = 65), and *bla*_OXA_ (30.4%, *n* = 32). Co-occurrence of ESBL genes was common, with *bla*_CTX-M//SHV/TEM/OXA_ and _*bla*CTX-M/TEM/SHV_ being the most frequent combinations, detected in 36% (n = 38) and 34% (*n* = 36) of isolates, respectively. Other combinations, such as *bla*_TEM/SHV_ and *bla*_TEM/CTX-M_, were less prevalent.

**Fig. 1:**
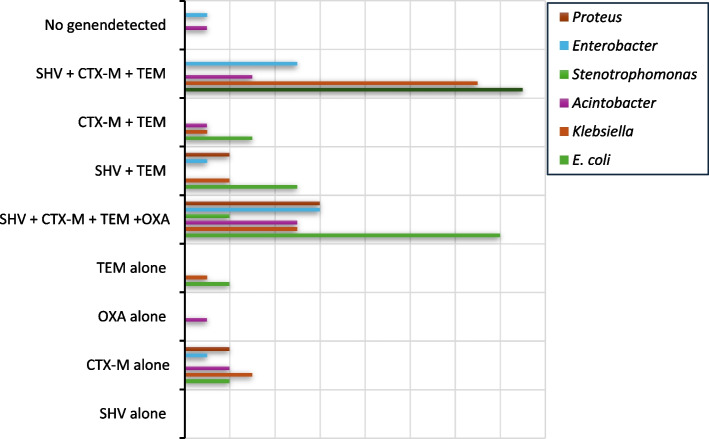
Distribution of ESBL genes across different bacterial species

The predominant ESBL gene was *bla*_CTX-M_, detected in 81% (*n *= 85) of isolates, followed by *bla*_TEM_ (62%, *n* = 74), *bla*_SHV_ (70.4%, *n* = 65), and *bla*_OXA_ (30.4%, *n* = 32). Co-occurrence of multiple ESBL genes was prevalent, with *bla*_CTX-M/SHV/TEM/OXA_ (36%, *n* = 38) and *bla*_CTX-M/TEM/SHV_ (34%, *n* = 36) representing the most frequent combinatorial profiles. Less prevalent combinations included *bla*_TEM/SHV_ and *bla*_TEM/CTX-M_. The analysis revealed that *Escherichia coli* predominantly harbored *bla*_CTX-M+TEM_, whereas *Proteus* spp. favored *bla*_SHV+CTX-M_. Notably, 12% (*n* = 13) of isolates exhibited single-gene carriage (e.g., *bla*_CTX-M_ or *bla*_TEM_) while two isolates lacked detectable ESBL loci, (Fig. 1). This genotypic heterogeneity underscores the adaptive diversity of ESBL dissemination within clinical pathogens.

### Antibiotic profile

Antimicrobial susceptibility profiling revealed meropenem (72.5% susceptibility) and amikacin (62.5%) as the most effective agents against ESBL-producing isolates, while amoxicillin (2.1%) and cephalexin (10.9%) demonstrated minimal efficacy. ESBL producers exhibited elevated resistance to cefotaxime (89.5%), ceftriaxone (70%), and ceftazidime (81.5%). Conversely, non-ESBL isolates retained higher sensitivity to meropenem (93.3%) and amikacin (83%), with maximal resistance observed for amoxicillin (87.8%) and co-trimoxazole (68.2%). Multidrug resistance (MDR; resistance to ≥ 4 agents) was prevalent in ESBL producers (89.5%, 94/105) compared to non-ESBL strains (34.2%, 105/307), underscoring the association between ESBL phenotype and pan-resistance evolution.

### Analysis of MDR, XDR, and PDR in hospital departments

The distribution of multidrug-resistant (MDR), extensively drug-resistant (XDR), and pan-drug-resistant (PDR) isolates across different hospital departments was analyzed. To determine if there were significant differences in the incidence of MDR, XDR, and PDR isolates among the different hospital departments, a chi-square test was performed. The results showed that the ICU had a significantly higher incidence of MDR, XDR, and PDR isolates compared to the medical and surgical wards (p < 0.05). The results of the ICU revealed the highest prevalence of MDR (85%), XDR (30%), and PDR (5%) strains. The Medical Ward followed with 70% MDR, 25% XDR, and 2% PDR, while the Surgical Ward revealed slightly lower resistance rates at 65% MDR, 20% XDR, and 3% PDR. In contrast, the outpatient clinic reported the lowest resistance rates with 55% MDR, 15% XDR and no observed resistance in PDR strains **(**Table 4**)**.

**Table 4:** Distribution of MDR, XDR, and PDR Isolates

Hospital Department	MDR (%)	XDR (%)	PDR (%)
Intensive Care Unit (ICU)	85	30	5
Medical Ward	70	25	2
Surgical Ward	65	20	3
Outpatient Clinic	55	15	0

### Statistical analysis

Based on the chi-square test, there is a highly significant association between the hospital setting, sample type, and the incidence of extended-spectrum β-lactamase (ESBL)-producing isolates (p < 0.0001). This suggests that the distribution of ESBL-positive isolates is not random and is influenced by the hospital setting and the type of sample collected.

## Discussion

The indiscriminate use of broad-spectrum antibiotics is a primary driver of multidrug resistance in bacteria and a significant contributor to the global incidence of extended-spectrum β-lactamases (ESBLs), which are responsible for life-threatening infections. The acquisition of *bla*_CTX-M_, *bla*_SHV_, *bla*_TEM_, and *bla*_OXA_ genes by Gram-negative bacteria in community and healthcare-associated infections has exacerbated the challenge of treating these infections with β-lactam antibiotics [14, 15].

In the present study, we observed that ESBL-producing Gram-negative bacteria isolated from various clinical specimens, including blood, respiratory samples, pus, urine, and CSF, harbored multiple ESBL genes (*bla*_CTX-M_, *bla*_TEM_, *bla*_OXA,_ and *bla*_SHV_). This finding is consistent with previous studies conducted in different countries [11, 16, 17]. Respiratory samples also contributed a notable number of isolates, highlighting the role of respiratory tract infections in spreading ESBL-positive bacteria.

In the ICU, patients with systemic infections are often treated with a wide range of antibiotics, which can contribute to the selection and spread of resistant bacteria [18]. These strains are likely transmitted through the air in healthcare settings, particularly in the ICU, between patients on mechanical ventilation [19]. This transmission route is supported by the fact that 37% of the samples in our study were from respiratory specimens, a finding that aligns with previous research in both developing and developed countries [2]. However, our results contrast with those of a study conducted in a different part of world reported a lower incidence of ESBL-producing Gram-negative bacteria with multiple ESBL genes in ICU nosocomial infections [20, 21]. This discrepancy may be attributed to differences in geographical regions, healthcare settings, or patient populations.

Notably, genotypic characterization demonstrated that *bla*_CTX-M_ was the predominant ESBL gene, constituting 85.3% of *Escherichia coli* and 84% of *Klebsiella* spp. isolates, frequently co-occurring with *bla*_TEM_. The *bla*_TEM_ variant ranked second, detected in 56% of *E. coli* and 88% of *K. pneumoniae* isolates [9, 22, 23], with prevalence rates of 57% and 46% in *Enterobacter* spp. and *Proteus* spp., respectively, followed by *bla*_SHV_. These findings align with global surveillance data from the Americas, Europe, Asia, Africa, and the Middle East [19]. Two isolates exhibited no amplifiable resistance loci, implying non-enzymatic (e.g., porin loss) or efflux-mediated resistance mechanisms. The genomic diversity observed highlights the multifactorial nature of ESBL dissemination, necessitating combinatorial phenotypic-genotypic surveillance frameworks to inform targeted antimicrobial regimens and reinforce infection control policies in multidrug-resistant pathogen management.

The global spread of the CTX-M-β-lactamase enzyme indicates its high incidence among ESBL-producing bacteria [24]. Studies conducted in Nigeria [9, 21, 25] further support the widespread occurrence of the *bla*_CTX-M_ gene in this region. Our findings are consistent with previous studies conducted in various regions, including Nepal and India [6, 26]. In these studies, *bla*_CTX-M_ and *bla*_TEM_ genes were commonly identified in ESBL-positive *E. coli* and *K. pneumoniae*. The incidence of *bla*_CTX-M_ has remained relatively high in these regions, despite variations in detection techniques employed [9, 22, 24, 25, 27].

The CTX-M-15 variant is often encoded by conjugative epidemic plasmids, such as IncFII, which contribute to its widespread dissemination [2, 14–21, 24, 25, 28, 29]. However, the relative incidence of different ESBL genes can vary depending on geographical location and temporal factors. For instance, in a Chinese study, the TEM gene predominated over SHV, while in Canada, SHV was the most prevalent ESBL gene. In India, the incidence of TEM genes declined over time, giving way to CTX-M genes [15, 17–19, 28]. These observations highlight the dynamic nature of ESBL gene incidence and the importance of ongoing surveillance to monitor changes in antibiotic resistance patterns across different regions.

*Acinetobacter baumannii* has emerged as a global healthcare challenge, particularly in hospital settings. Our study found that *A. baumannii* accounted for 13% of bacterial isolates, with a higher incidence in the ICU (10%) compared to outpatient clinics (3%). One of the most significant resistance mechanisms of *A. baumannii* strains is the production of ESBLs. Previous studies have reported a high incidence of ESBL-producing *A. baumannii* strains worldwide [27, 30]. In our investigation, 61.5% of ESBL-positive *A. baumannii* strains harbored the *bla*_OXA_ gene, along with *bla*_SHV_, *bla*_TEM_, and *bla*_CTX-M_ genes [21].

*Acinetobacter* species are known for their high incidence of carbapenemase-encoding genes, including *bla*_OXA_. Our study confirmed this, with *bla*_OXA_ being the most frequently detected gene among *Acinetobacter* isolates (92%). These variants have been previously isolated from various regions, including France, Spain, and Turkey, indicating their global spread [21]. In a previous study by Zarabadi et al., 83.6% of *A. baumannii* isolates were ESBL producers, with *bla*_TEM_ being the most prevalent ESBL gene (82–87%), followed by *bla*_SHV_ (67–78%) [16]. Our findings align with these observations, as 61.5% of our isolates carried *bla*_SHV_, *bla*_TEM_, *bla*_CTX_, and *bla*_OXA_ genes.

While our study found a high incidence of *bla*_TEM_ and *bla*_SHV_ genes in *A. baumannii*, other studies have reported varying frequencies of these genes. For instance, a previous study identified lower frequencies of *bla*_TEM_ (4.3%) and *bla*_SHV_ (43.4%) in *A. baumannii* strains isolated from burn wound infections [21]. These discrepancies highlight the potential variability in ESBL gene incidence and types among *A. baumannii* strains in different geographical regions and clinical settings.

The investigation revealed the prevalence of *Stenotrophomonas maltophilia* in (2% *n* = 2) of clinical isolates analyzed. Notably, one isolate exhibited pan-β-lactam resistance, demonstrating insensitivity to all tested β-lactam antimicrobial agents, while retaining susceptibility to trimethoprim-sulfamethoxazole (TMP-SMX). *S. maltophilia*, recognized as an opportunistic MDR pathogen of increasing clinical concern, is notorious for its intrinsic resistance mechanisms and capacity to cause nosocomial infections, presenting a considerable therapeutic challenge in immunocompromised populations and healthcare-associated environments.

In this study, we also described different antibiotic resistance genes incidence found in the isolates originating from hospitalized and outpatient wards, which may reflect different usage of antimicrobials in the community compared to the hospital [6, 7, 14, 27]. The epidemiological significance of outpatient-derived isolates may be postulated to mirror the prevailing microbial ecology within community reservoirs. Individuals acquiring infections in community settings frequently engage in self-medication practices driven by limited access to clinical oversight, resulting in empiric exposure to narrow-spectrum antimicrobial agents accessible via non-prescription channels in commercial pharmaceutical outlets. This behavioral pattern fosters selective pressure from non-targeted antimicrobial use, potentially amplifying resistance determinants within community-acquired pathogens and complicating empirical treatment models.

The observed clinical epidemiology suggests that antimicrobial resistance (AMR) phenotypes necessitating hospitalization are predominantly those exhibiting resistance to community-accessible empiric therapies. Consequently, outpatient-acquired infections demonstrate homogenized AMR profiles, reflecting selective pressures from limited, over-the-counter antimicrobial exposure. In contrast, the increased genotypic and phenotypic diversity of resistance determinants in inpatient isolates likely correlates with institutional exposure to broader-spectrum antimicrobials, multidrug therapeutic regimens, and prolonged empirical treatment protocols in nosocomial settings.

The predominance of extended-spectrum β-lactamase (ESBL)-producing isolates harboring co-localized resistance genes (*bla*_CTX-M_, *bla*_TEM_, *bla*_SHV_) raises significant therapeutic concerns, as this genetic architecture may drive persistent β-lactam resistance despite β-lactamase inhibitor adjuvants (e.g., clavulanic acid) [29]. This phenomenon may arise from enzymatic hyperproduction, wherein elevated β-lactamase expression saturates inhibitor capacity, coupled with efflux pump activity or permeability alterations. From an infection prevention perspective, the plasmid-mediated horizontal gene transfer (HGT) of MDR cassettes necessitates the stringent implementation of antimicrobial stewardship programs (ASPs), enhanced environmental decontamination protocols, and active surveillance for carbapenem-resistant Enterobacterales (CRE) or ESBL colonization. Containment of these mobile genetic elements is critical to curbing nosocomial transmission networks and preserving last-line antimicrobial efficacy in high-risk clinical populations.

## Conclusion

This study demonstrates the substantial burden of ESBL-producing Enterobacterales within our hospital, necessitating the urgent implementation of evidence-based infection control protocols. Critical interventions include antimicrobial stewardship programs (ASPs) to restrict inappropriate antibiotic use, rigorous hand hygiene compliance, and targeted environmental disinfection to disrupt transmission reservoirs. Future research must prioritize elucidating epidemiological risk factors for ESBL acquisition, investigating transmission dynamics through molecular surveillance, and developing innovative therapies such as β-lactamase inhibitor adjuvants and novel antimicrobial combinations. These strategies are critical to mitigate the proliferation of multidrug-resistant pathogens and align with global antimicrobial resistance (AMR) containment initiatives.

### Limitations

The fact that we were unaware of the patients'prior antibiotic treatments before sample collection may have contributed to the selection of MDR bacteria, which is one drawback of our study. We only employ primers for particular alleles of the *bla*_CTX-M_, *bla*_SHV_, and *bla*_TEM_ genes, which is another constraint. The strains under study may have other variations in these resistance genes that our methods missed. The study was conducted in a single hospital and might not reflect the broader picture of MDR incidence in the region.

## Supplementary Information

Supplementary Material 1.

## Data Availability

Our data supporting the findings of this study are available within the article and its supplementary materials. Additional data are available from the corresponding author upon reasonable request.

## Abbreviations

ESBLs: Extended-Spectrum β-Lactamase Genes
MDR: Multidrug-resistant
XDR: Extensively drug-resistant
PDR: Pan-drug-resistant
CRE: Carbapenem-resistant enterobacterales
