# Supplementary material for: Incidence of Extended Spectrum β-Lactamase Genes (ESBLs) among community and health care infection in Mansoura University Hospital, Egypt
Source: BMC Microbiol. 2025 May 22;25:316. doi: 10.1186/s12866-025-04030-3 (PMC12096576; doi:10.1186/s12866-025-04030-3)
Supplement: Supplementary file 1 — Supplementary Material 1. [file 12866_2025_4030_MOESM1_ESM.pdf]

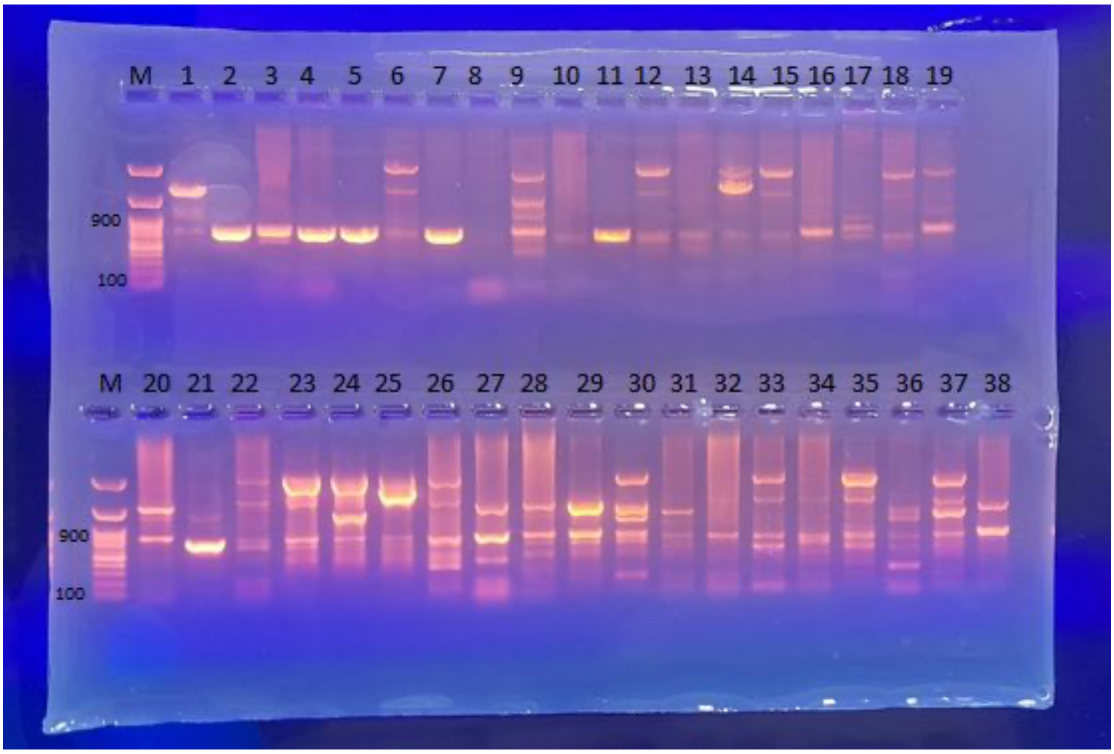

Supplementary Figure S1: Detection of bla_{OXA} gene (Lanes 1-38; 4, 708 bp) in clinical isolates using conventional PCR. Lane M refers to 100 bp DNA ladder (BIO-HELIX).

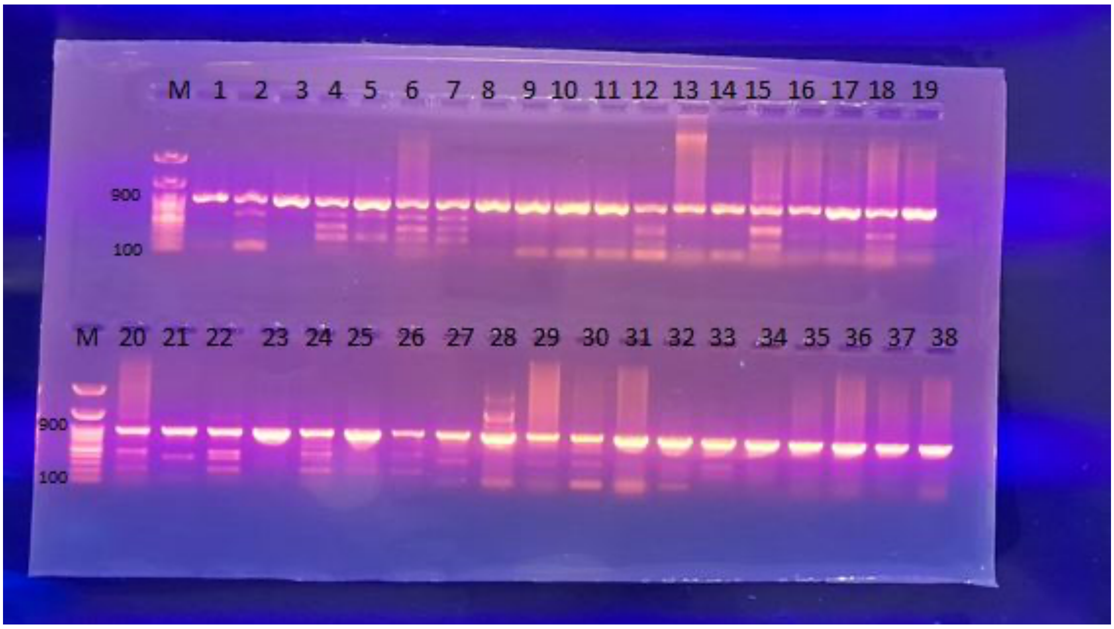

Supplementary Figure S2: Detection of bla_{TEM} gene (Lanes 1-34; 3, 931 bp) and $bla_{CTX\text{-}M}$ gene (Lanes 35-38; 909 bp) in clinical isolates using conventional PCR. Lane M refers to 100 bp DNA ladder (BIO-HELIX).

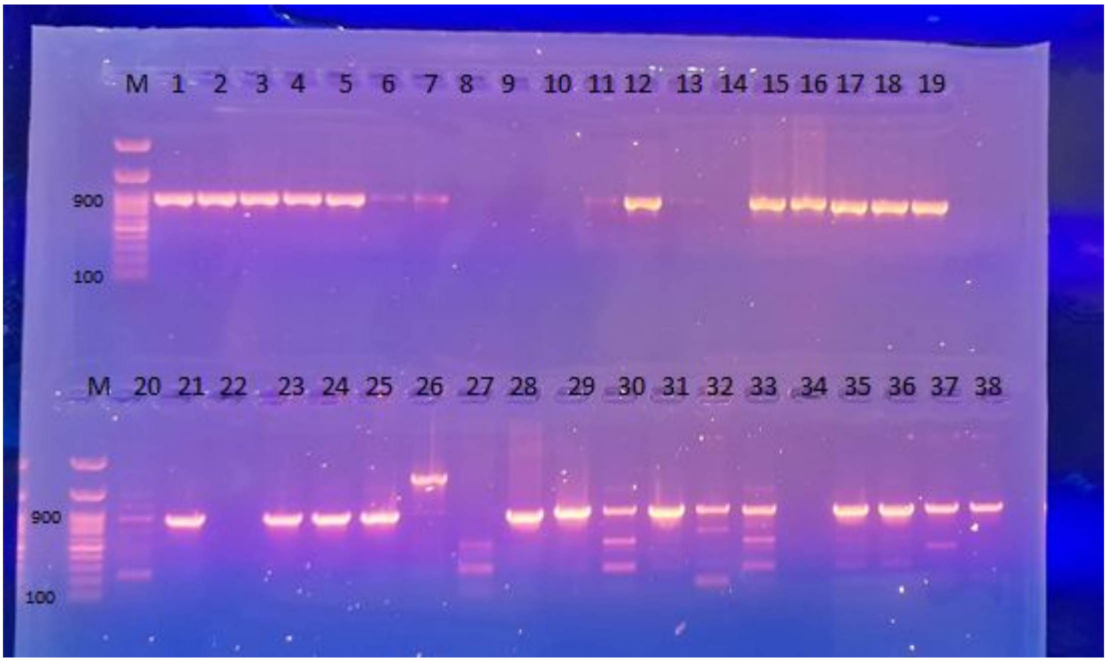

Supplementary Figure S3: Detection of *bla*$_{CTX-M}$ gene (Lanes 1-16; 909 bp), *bla*$_{SHV}$ gene (Lanes 17-28; 868 bp) and *bla*$_{TEM}$ (Lanes 29-38; 3, 931 bp) using conventional PCR. Lane M refers to 100 bp DNA ladder (BIO-HELIX).
